# Evaluating the development and well-being assessment (DAWBA) in pediatric anxiety and depression

**DOI:** 10.1186/s13034-023-00696-7

**Published:** 2024-01-20

**Authors:** Paia Amelio, Chase Antonacci, Parmis Khosravi, Simone Haller, Katharina Kircanski, Erin Berman, Lisa Cullins, Krystal Lewis, Mollie Davis, Chana Engel, Kenneth Towbin, Argyris Stringaris, Daniel S. Pine

**Affiliations:** 1 Emotion and Development Branch, National Institute of Mental Health, National Institutes of Health, Bethesda, MD USA; 2 Divisions of Psychiatry and Psychology and Language Science, University College London, London, UK; 3 National and Kapodistrian University of Athens, Athens, Greece

**Keywords:** DAWBA, Anxiety, Depression, Internalizing, Cognitive behavioral therapy, Psychiatric interview

## Abstract

Enhancing screening practices and developing scalable diagnostic tools are imperative in response to the increasing prevalence of youth mental health challenges. Structured lay psychiatric interviews have emerged as one such promising tool. However, there remains limited research evaluating structured psychiatric interviews, specifically their characterization of internalizing disorders in treatment-seeking youth. This study evaluates the relationship between the Development and Well-Being Assessment (DAWBA), a structured psychiatric interview, and established measures of pediatric anxiety and depression, including the Screen for Child Anxiety Related Disorders (SCARED), the Pediatric Anxiety Rating Scale (PARS), and the Mood and Feelings Questionnaire (MFQ). The study comprised two independent clinical samples of treatment-seeking youth: sample one included 55 youth with anxiety and 29 healthy volunteers (HV), while sample two included 127 youth with Major Depressive Disorder and 73 HVs. We examined the association between the DAWBA band scores, indicating predicted risk for diagnosis, the SCARED and PARS (sample one), and the MFQ (sample two). An exploratory analysis was conducted in a subset of participants to test whether DAWBA band scores predicted the change in anxiety symptoms (SCARED, PARS) across a 12-week course of cognitive behavioral therapy. The results revealed that the DAWBA significantly predicted the SCARED, PARS and MFQ measures at baseline; however, it did not predict changes in anxiety symptoms across treatment. These findings suggest that the DAWBA may be a helpful screening tool for indexing anxiety and depression in treatment-seeking youth but is not especially predictive of longitudinal trajectories in symptomatology across psychotherapy.

**Supplementary Information:**

The online version contains supplementary material available at 10.1186/s13034-023-00696-7.

## Introduction

The rising prevalence of pediatric mental health issues underscores the importance of validated, scalable screening tools [1, 2]. Specifically, there is a need for structured psychiatric assessments that both can be administered by lay interviewers and are validated by established measures of psychopathology. Considering the high rates of mood and anxiety symptoms (prevalence of 25.1% for anxiety and 11.5% for depressive mood) in youth [1, 2], this need applies particularly to the domain of emotional problems. The current study evaluates the relationship between parent-, child-, and clinician-rated scales of internalizing symptoms and the Development and Well-Being Assessment (DAWBA), a widely used structured psychiatric interview [3].

Prior attempts to relate lay interviews to clinical assessments generate relatively poor psychometrics for emotional problems. One set of studies utilized the Diagnostic Interview Schedule for Children (DISC) [4], which exhibits acceptable reliability and validity combining youth and parent reports for externalizing disorders (test-retest kappa = 0.48–0.66, validity with retest kappa = 0.49–0.70, concurrent validity kappa = 0.65–0.80) [5]. However, relatively poor psychometrics exist for the DISC’s internalizing modules (test-retest kappa = 0.35–0.52; validity with retest kappa = 0.32–0.53; concurrent validity kappa = 0.37–0.57) [5] in which the DISC demonstrates poor to fair test-retest reliability and yields moderate concurrent validity across both anxiety and depression. Additional studies similarly report weak associations between the DISC and established self-report anxiety measures [6]. Other interviews and more recent versions of the DISC may generate superior psychometrics [7]; however, few studies have evaluated concordance between rating scales and structured psychiatric interviews in samples where the need may be greatest – that is, in youth seeking treatment for emotional problems.

The Screen for Child Anxiety Related Disorders (SCARED) is one widely used self- and parent-reported measure of anxiety spanning multiple diagnostic domains [8]. Likewise, the Mood and Feelings Questionnaire (short version; MFQ) is a self-report questionnaire assessing depressive symptoms [9]. The Pediatric Anxiety Rating Scale (PARS) is an established clinician-rated measure of anxiety severity and impairment [10]. While these assessments are useful for measuring internalizing psychopathology, the DAWBA offers a brief, widely distributable cross-disorder evaluation of youth psychopathology, an adaptive question structure, and more comprehensive integration of child and parent response data [3, 7]. The DAWBA has also been validated across several psychiatric disorders and generates independent ratings of mood and anxiety symptoms that may relate to other scales of internalizing psychopathology [3, 11–13]. Given the relevance and challenge of integrating reports from multiple sources [6], in the current study, we evaluate relations between these measures and band scores generated by the DAWBA that integrate response data from both caregivers and youth [14].

The DAWBA evaluates the probability of 19 psychiatric illnesses via an adaptive online questionnaire in which respondents are presented a unique series of questions for each disorder dependent upon previous responses [3]. The potential value of the DAWBA lies in both its scalability as a short computerized assessment and its integration of data across multiple respondents to predict risk for diagnosis [7, 11]. If the DAWBA’s psychometrics translate to populations of youth seeking treatment for emotional problems, the interview could enhance accessibility of psychiatric screening in multiple settings where resources are limited [15, 16]. In addition to screening, we assessed the DAWBA’s ability to predict treatment response. Previous clinical trials have demonstrated the sensitivity of the SCARED, MFQ, and PARS to symptom improvement in efficacious treatments [17–19]. Moreover, a recent review reported that baseline severity constitutes one of the strongest predictors of clinical trajectories [20]. In a study that combined measures of internalizing symptoms at baseline, findings indicated that low anxiety severity predicted better treatment outcomes after 12 weeks, as indexed by the PARS [21]. The SCARED has also been used to predict treatment response and remission across both parent and child reports [22]. Therefore, we aimed to determine whether the band scores generated by the DAWBA at baseline predict therapeutic response, as indexed by these established measures across treatment. If predictive of symptom trajectories in treatment-seeking youth, the DAWBA band scores might reasonably offer clinical utility beyond a baseline diagnostic assessment.

The current study tests the hypothesis that, first, DAWBA band scores are associated with self- and parent-reported measures of anxiety and depression and clinician-reported measures of anxiety. Second, we hypothesize that the DAWBA anxiety band scores collected at baseline will predict the change in SCARED and PARS scores over the course of treatment. Due to the lack of availability of MFQ data across treatment in the depression sample, we did not examine the DAWBA MDD band score in relation to depression symptom trajectories.

## Methods

### Participants

The study included a total of 284 youth (see Table 1 for sample characteristics). Eighty-four were part of one sample comprising 55 participants with an anxiety disorder and 29 healthy volunteers (i.e., anxiety sample). The remaining 200 youth were part of another sample from the NIH Characterization and Treatment of Depression Study [23], comprising 127 participants with Major Depressive Disorder (MDD) and 73 healthy volunteers (i.e., depression sample).

The 84 participants in the anxiety sample were interviewed by trained clinicians masked to all other data using the Kiddie Schedule for Affective Disorders and Schizophrenia for School-Aged Children – Present and Lifetime Version (K-SADS-PL) [24]. Fifty-five of these participants met the criteria for one or more of the following anxiety disorders: Generalized Anxiety Disorder (GAD), Separation Anxiety Disorder (Sep AD), or Social Anxiety Disorder (Social AD) (see Table 1 for sample haracteristics). The 29 healthy volunteers within the anxiety sample were free from any psychiatric diagnoses. Exclusion criteria for the anxiety sample can be found at https://clinicaltrials.gov/study/NCT00018057 (diagnostic criteria listed in supplement).

In the depression sample, participants were interviewed by trained clinicians using the K-SADS-PL [24] who were not masked to scores on the DAWBA. Depressed participants met criteria for Major Depressive Disorder (MDD) while healthy volunteers were free from any psychiatric diagnoses (see Table 1 for sample characteristics). Exclusion criteria for the depression sample can be found at https://www.clinicaltrials.gov/study/NCT03388606 (diagnostic criteria listed in supplement).

### Measures

#### Development and well-being assessment (DAWBA)

The Development and Well-Being Assessment (DAWBA) is a package of questionnaires, interviews, and rating techniques designed to generate Diagnostic and Statistical Manual of Mental Disorders (DSM-5) and International Classification of Diseases (ICD-10) psychiatric diagnoses for youth ages 11–17. The DAWBA was collected using an online platform developed by Youth in Mind [14], with all participants completing the self-report and their guardian completing the parent-report. For all participants, the online DAWBA assessment began with the Strengths and Difficulties Questionnaire (SDQ), a brief screening evaluation consisting of twenty-five questions that span five scales of behavioral difficulty: emotional symptoms (5 questions), conduct problems (5 questions), hyperactivity/inattention (5 questions), peer relationship problems (5 questions), and prosocial behavior (5 questions). Following the SDQ, participants were presented screening questions for each of 19 psychiatric disorder modules. For depression and anxiety modules, the DAWBA queries about symptoms ranging from the prior four weeks to six months. If the participant responds affirmatively to either the disorder-related SDQ question or one of the disorder-specific screening questions, meeting the set symptom threshold, the DAWBA will then prompt the individual to answer further explicating questions. However, if the participant does not meet the set symptom threshold, the DAWBA will bypass the remaining items for that disorder module.

*DAWBA Band Scores*. For all participants completing the DAWBA, three band scores are generated for each disorder module, representing the parent, child, and combined parent-child predictions of risk for a disorder. Band scores are integer values ranging from 0 to 5 indicating the following probabilities of a child meeting diagnostic criteria: “0” (*p* < 0.1%), “1” (*p* ~ 0.5%), “2” (*p* ~ 3%), “3” (*p* = 15%), “4” (*p* = 50%), and “5” (*p* ≥ 70%). For all analyses presented in the results section, we used the DAWBA combined (parent and child) band scores based on DSM-5 criteria, which is generated by the DAWBA’s computer algorithms [11]. Previous reports have also derived DAWBA symptom scales, which combine data from various disorders and offer a continuous measure of symptom experience [25]; see the supplement for analyses using symptom scales.

#### Screen for child anxiety related emotional disorders (SCARED)

The SCARED is a widely used child- and parent-reported instrument to assess childhood anxiety disorders including GAD, Sep AD, Panic Disorder, and Social AD [8]. Both the parent and child questionnaires comprise 41 items spanning five DSM-4 domains. The SCARED possesses moderate parent-child agreement and good internal consistency (intraclass correlation coefficients = 0.37–0.62; α = 0.7–0.9), discriminant validity, and test-retest reliability (ρ = 0.6–0.9) [26]. The SCARED generates a composite score, one from the parent assessment and one from the child assessment; in our analysis, we averaged these two scores to calculate a combined parent- and child-reported SCARED value (SCARED-CP) [27] to allow for comparison with the DAWBA’s combined parent-child band scores. Greater SCARED scores indicate more severe anxiety symptom presentation. Participants who completed the SCARED more than three months from the completion of the DAWBA were excluded from all analyses using SCARED data. The three-month cutoff allowed for a sufficient sample size across various measures while also excluding overt outliers. In an exploratory analysis, we also tracked anxious participants’ SCARED scores across treatment, using their baseline score collected at screening along with successive scores collected at predefined intervals during cognitive behavioral therapy (CBT): pre-exposure (week 3 of treatment), mid-exposure (week 8 of treatment), and post-exposure (week 12 of treatment).

#### The mood and feelings questionnaire (MFQ)

The MFQ provides an assessment of depression symptom severity and generates a composite score from both child and parent reports [9]. As with the SCARED, these composite scores were averaged together in our analyses to provide a combined parent and child MFQ score (MFQ-CP) [28] in order to assess the relationship with the DAWBA’s combined parent-child band score. Within the MDD sample, the MFQ was collected from both depressed and healthy volunteers at baseline when participants were enrolled in the study; this baseline score was used in all analyses. Each of the 13 items was scored and summed to generate a composite score ranging from 0 to 26, with greater scores indicating more severe depression or mood disorder presentation. For participants who completed the MFQ more than three months before or after the completion of the DAWBA, their data were omitted from the respective analyses.

#### Pediatric anxiety rating scale (PARS)

The PARS is a clinician-rated assessment of anxiety symptoms and is widely used in treatment studies [10, 17]. When administered, both child and parent responses are considered in the clinician’s assessment, resulting in a combined overall score. A higher score indicates more severe anxiety symptom presentation. PARS was collected at the screening visit and at three time points during treatment: CBT week 3, CBT week 8, and CBT week 12. Youth who completed the DAWBA and PARS within three months of each other were included in analyses of PARS data.

### Procedure

Written informed consent and assent were obtained from the guardian(s) and the child, respectively. All procedures were approved by the NIMH Institutional Review Board, and all participants were compensated for participation. All data presented in this study were collected from children and their guardian(s) as part of larger treatment studies of children with mood or anxiety disorders. DAWBA was administered at screening in both groups. However, as noted above, in the anxiety sample, clinicians were masked to the results of the DAWBA when assigning a diagnosis via the K-SADS-PL; in contrast, clinicians performing the K-SADS-PL in the depression sample were not masked to the DAWBA.

The relationship between the DAWBA and established self-report instruments was evaluated within the two samples. In the anxiety cohort, both SCARED-CP and PARS were administered at the screening visit and across treatment at the intervals defined above. In the depression sample, the MFQ-CP was administered at screening. All questionnaire data collected more than three months from the DAWBA were excluded in both the anxiety and depression samples. In both samples, if participants completed repeated symptom measures within three months of the DAWBA, we used data temporally closest to the collection of the DAWBA.

### Statistical analysis

All statistical analyses were performed in RStudio (version 2022.07.1).

#### 1a and 1b – self-report measures: SCARED-CP and MFQ-CP

A linear regression analysis was performed to assess whether the three DAWBA band scores for anxiety disorders (GAD, Sep AD, and Social AD) predicted the SCARED-CP. Similarly, a linear regression was performed in the depression sample between the DAWBA MDD band score and MFQ-CP.

#### 1c – SCARED-CP across treatment

To assess whether the DAWBA predicted treatment outcomes, we calculated the difference score for the SCARED-CP by subtracting the pre-treatment (baseline) score from the post-treatment (CBT week 8 or CBT week 12) score. A linear regression analysis was performed using the DAWBA band scores to predict the SCARED-CP difference score. In calculating the difference score, we used the latest SCARED-CP score a subject completed, either from week 8 or 12 of CBT.

#### 2a – clinician-report measure: PARS

A comparable approach as outlined in Statistical Analyses 1a was used for PARS in which linear regression was used to predict the PARS score from the DAWBA anxiety band scores (GAD, Sep AD, and Social AD) in the anxiety sample.

#### 2b – PARS across treatment

A comparable approach as outlined in Statistical Analysis 1c was used for PARS, in which we performed a linear regression analysis to predict the PARS difference score (post-treatment - pre-treatment) from the DAWBA band scores (GAD, Sep AD, and Social AD). In calculating the difference score, we used the latest PARS a subject completed, either from week 8 or 12 of CBT.

**Table 1:** Demographic characteristics of sample

	*Sample 1: Anxiety* *N = 84*				*Sample 2: Depression* ^*a*^ *N = 200*			
Baseline characteristic	Anxious *n* = 55		Healthy Volunteers *n* = 29		Depressed *n* = 127		Healthy Volunteers *n =* 73	
	M	SD	M	SD	M	SD	M	SD
Age	11.96	3.00	12.34	2.54	15.35	1.39	14.82	1.56
	***n***	**%**	***n***	**%**	***n***	**%**	***n***	***%***
Sex								
Women	39	70.9	18	62.1	87	68.5	45	61.64
Men	16	29.1	11	37.9	40	31.5	28	38.36
Ethnicity								
Hispanic/Latinx	2	3.63	0	0	11	8.66	5	6.85
Not Hispanic/Latinx	48	87.3	29	100	115	90.55	68	93.15
Unknown	5	9.1	0	0	1	0.79	0	0
Race								
White	33	60.0	16	55.17	82	64.57	47	64.38
Black/African American	4	7.27	1	3.45	14	11.02	9	12.33
Asian American	3	5.45	3	10.34	11	8.66	10	13.7
Pacific Islander/Native Hawaiian	-	-	-	-	1	0.79	1	1.37
Multiple Races	11	20	9	31.03	17	13.39	5	6.85
Unknown	4	7.27	0	0	2	1.57	1	1.37
Mood/Anxiety Disorders ^b^								
Depression	0	0	0	0	127	100	0	0
Generalized Anxiety Disorder	40	85.1	0	0	-	-	-	-
Separation Anxiety	22	47.8	0	0	-	-	-	-
Social Phobia	20	42.6	0	0	-	-	-	-
**Measures**	**M**	**SD**	**M**	**SD**	**M**	**SD**	**M**	**SD**
SCARED-CP ^c^	34.34	13.19	7.44	3.75				
Parent	30.6	13.51	5.3	2.94	-	-	-	-
Child	35.44	16.52	9.77	7.02	-	-	-	-
PARS ^d^	14.47	3.83	1.62	2.5	-	-	-	-
MFQ-CP ^c^					13.15	5.02	0.89	1.77
Parent	-	-	-	-	11.84	6.21	0.50	1.08
Child	-	-	-	-	14.43	6.11	1.27	3.33

^a^ The depression sample is from the NIH Characterization and Treatment of Depression Study ^23^

^b^ Anxiety diagnoses were determined by clinician assessment via the K-SADS-PL. Depression diagnoses were based on clinician-assessed “Participant Type” via the K-SADS-PL since diagnosis could have changed from *sub-MDD* subthreshold Major Depressive Disorder to *MDD* Major Depressive Disorder throughout research and treatment; *SCARED* Screen for Child Anxiety Related Emotional Disorders; *PARS* Pediatric Anxiety Rating Scale; *MFQ* Mood and Feelings Questionnaire

^c^ The number of subjects varied across analyses; the respective N for each analysis is reported in the text

^d^ Symptom levels based on clinician-report measures

## Results

### STUDY 1: relationship between DAWBA and established self-report instruments

#### 1a – self-report measures: SCARED-CP

**Fig. 1:**
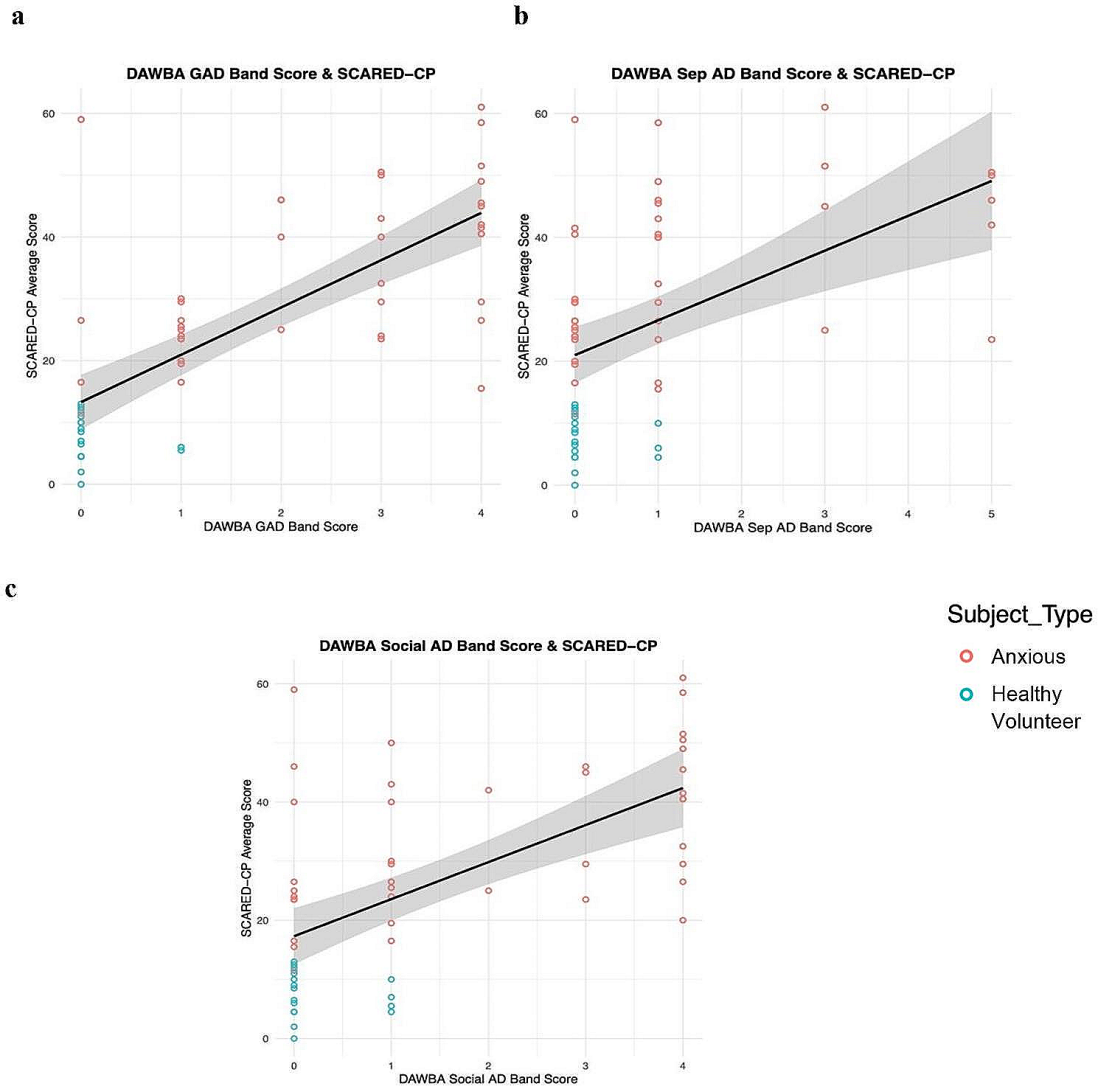
(**a**) Correlation between DAWBA Generalized Anxiety Disorder (GAD) band score and the combined Screen for Child Anxiety Related Disorders – Child and Parent Report (SCARED-CP) across both anxious patients and healthy volunteers. (**b**) Correlation between DAWBA Separation Anxiety Disorder (Sep AD) band score and the combined SCARED-CP across both anxious patients and healthy volunteers. (**c**) Correlation between DAWBA Social Anxiety Disorder (Social AD) band score and the combined SCARED-CP across both anxious patients and healthy volunteers. Healthy volunteers = blue; anxious patients = red

The results indicate that the DAWBA band scores for each anxiety disorder (GAD, Sep AD, and Social AD) were significantly (Table 2) and weakly-to-moderately associated with the SCARED-CP (n = 57; 40 Anxious, 17 HVs; Fig. 1). The mean number of days between the collection of the SCARED-CP and the DAWBA was 16.13 (SD = 13.77, range: 0–62 days).

**Table 2:** Self-report and clinician-report regressions

Predictor	*b*	τ	*df*	*t*	*p*	CI
***SCARED-CP***						
DAWBA GAD	7.65	0.61	55	8.02	< 0.001	[5.74, 9.56]
DAWBA Sep AD	5.63	0.42	55	5.63	< 0.001	[3.04, 8.21]
DAWBA Social AD	6.27	0.48	55	5.70	< 0.001	[4.06, 8.47]
***MFQ-CP***						
DAWBA MDD	3.23	0.68	192	24.00	< 0.001	[2.97, 3.50]
***PARS***						
DAWBA GAD	3.26	0.69	59	9.36	< 0.001	[2.57, 3.96]
DAWBA Sep AD	1.59	0.35	58	2.94	0.005	[0.51, 2.67]
DAWBA Social AD	2.42	0.51	58	5.51	< 0.005	[1.54, 3.29]

τ: Kendall tau; *df*: degrees of freedom; CI: 95% confidence interval [upper bound, lower bound]

Significance levels at α < 0.05

#### 1b – self-report measures: MFQ-CP

**Fig. 2:**
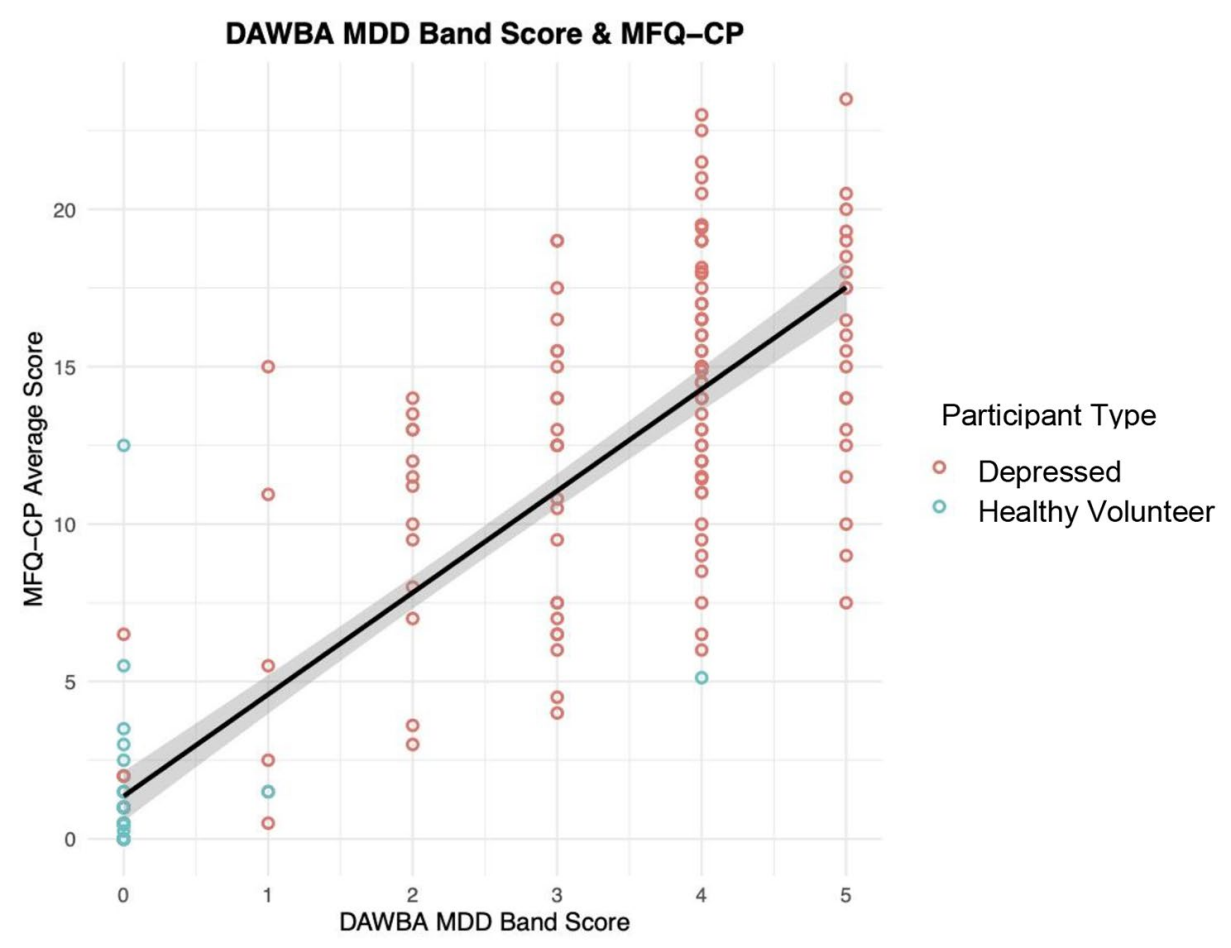
Correlation between DAWBA Major Depressive Disorder (MDD) band score and the Mood and Feelings Questionnaire – Child and Parent Report (MFQ-CP) across both depressed patients (red) and healthy volunteers (blue)

The DAWBA MDD band score significantly (Table 2) and strongly predicted the MFQ-CP score (n = 194, 124 Depressed, 70 HVs; Fig. 2). The mean number of days between the collection of the MFQ-CP and the DAWBA was 36.41 (SD = 19.95, range: 0–91 days).

#### 1c – SCARED-CP across treatment

**Fig. 3:**
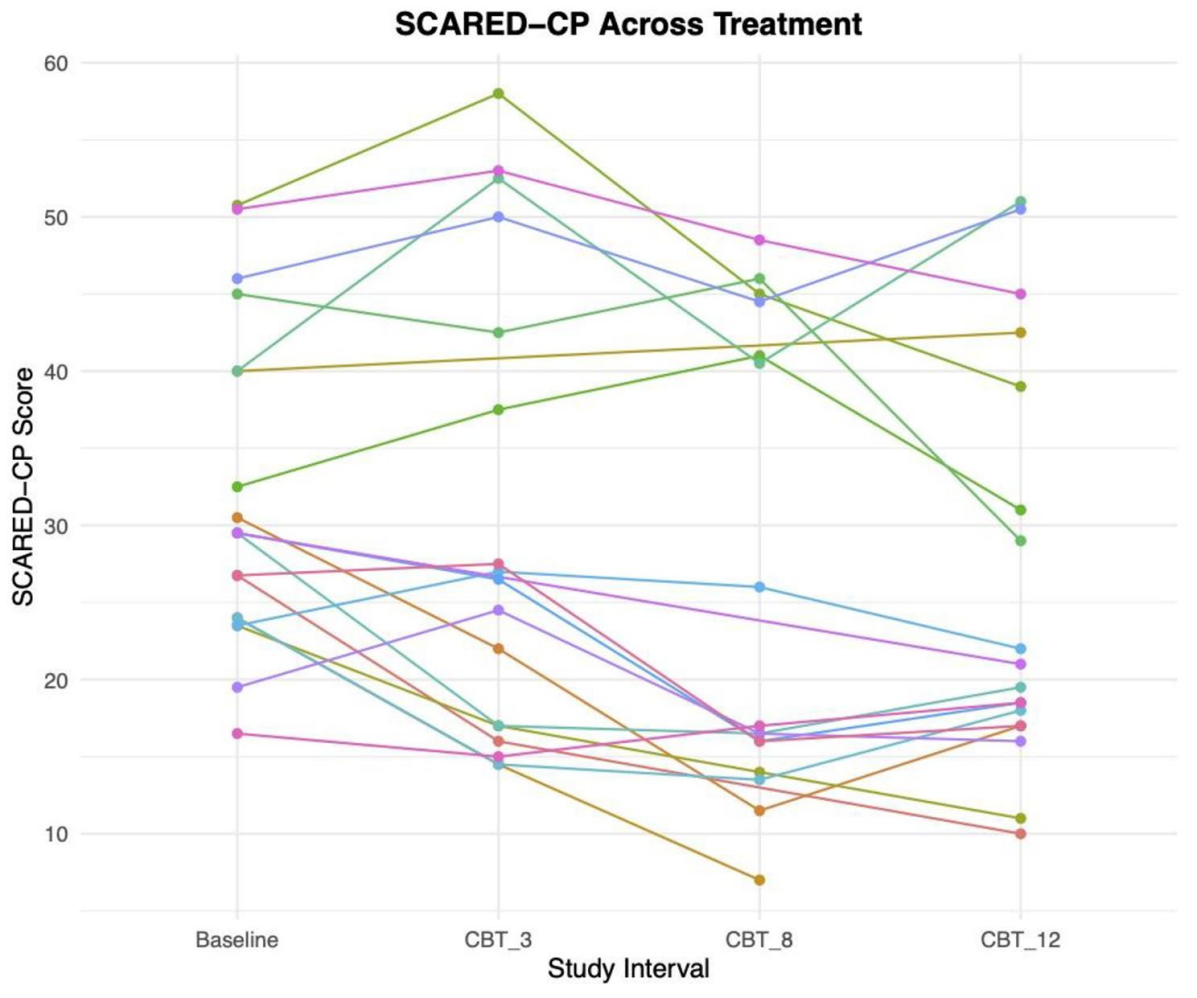
Lines indicate individual participants’ Screen for Child Anxiety Related Disorders – Child and Parent (SCARED-CP) score mapped at each study interval throughout the treatment period. Beginning at baseline and ending at week 12 of cognitive behavioral therapy (CBT), the SCARED-CP was collected four times during the study for anxious participants in treatment. A downward trend would indicate a reduced symptom severity observed by parent/experienced by child

Across treatment, the mean change in SCARED-CP score was − 6.78 (SD = 7.98). The DAWBA anxiety band scores (GAD, Sep AD, and Social AD) at baseline (n = 19) did not significantly predict the SCARED-CP difference score (Table 2), which captures the change in anxiety symptom severity across treatment (Fig. 3). There were no significant results (*p*s > 0.70). Similarly, the baseline SCARED-CP did not predict the difference score (*p* = 0.56).

### STUDY 2: relationship between DAWBA and established clinician-rated instrument of anxiety

#### 2a – clinician-report measure: PARS

**Fig. 4:**
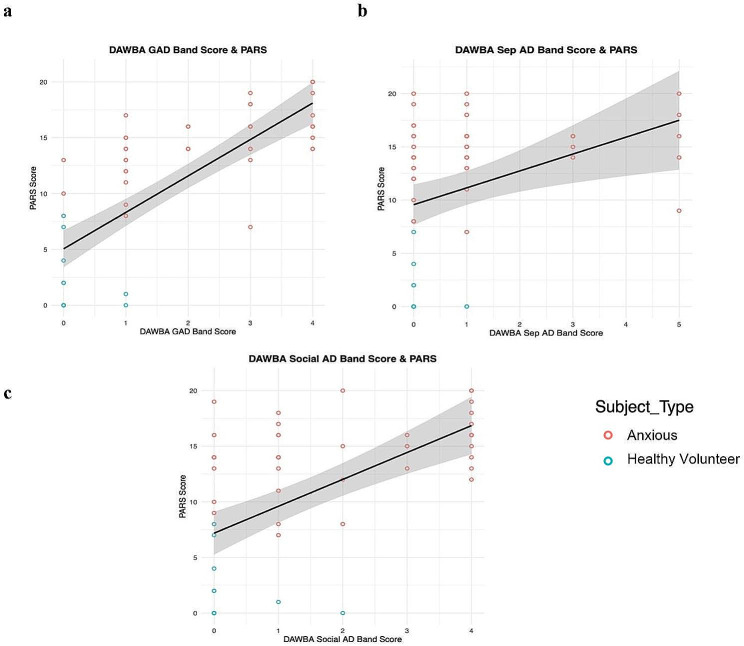
(**a**) Correlation between DAWBA Generalized Anxiety Disorder (GAD) band score and the combined parent and child report from the Pediatric Anxiety Rating Scale (PARS) across both anxious patients and healthy volunteers. (**b**) Correlation between DAWBA Separation Anxiety Disorder (Sep AD) band score and the combined parent and child report from the PARS across both anxious patients and healthy volunteers. (**c**) Correlation between DAWBA Social Anxiety Disorder (Social AD) band score and the combined parent and child report from the PARS across both anxious patients and healthy volunteers. Healthy volunteers = blue; anxious patients = red

Our results indicate that the DAWBA band scores for each anxiety disorder (GAD, Sep AD, and Social AD) significantly (Table 2) predicted PARS scores (n = 63, 46 Anxious, 17 Healthy Volunteers; Fig. 4). The mean number of days between the completion of the DAWBA and PARS was 17.27 (SD = 20.04, range: 0–90 days).

#### 2b – PARS across treatment

**Fig. 5:**
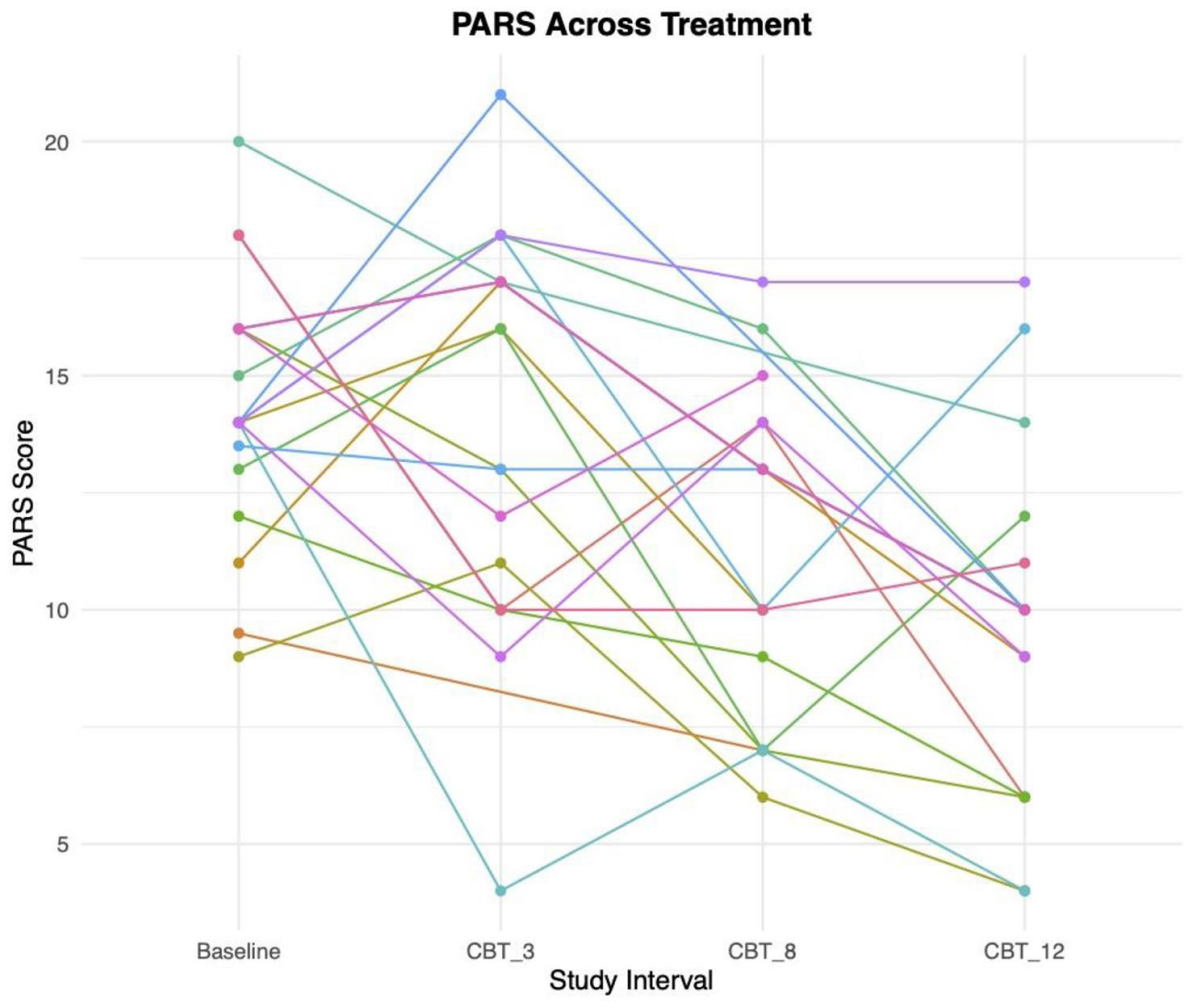
Lines indicate individual participants’ Pediatric Anxiety Rating Scale (PARS) scores mapped at each study interval throughout the treatment period. Beginning at baseline and ending at week 12 of cognitive behavioral therapy (CBT), the PARS was collected four times during the study for anxious participants in treatment. A downward trend would indicate a reduced symptom severity observed by clinicians

Across treatment, the average change in PARS score was − 4.55 (SD = 3.75). The DAWBA band scores (GAD, Sep AD, and Social AD) at baseline did not significantly (Table 2) predict the PARS difference score across treatment (n = 20; Fig. 5); *p*s > 0.09, nor did the baseline PARS score predict the difference score (*p* = 0.10).

## Discussion

This study examined associations between the DAWBA and established measures of anxiety and depression at baseline and across treatment. Three main findings arose from the study. First, the DAWBA band scores significantly predicted both self- and parent-reported measures (SCARED-CP, MFQ-CP) in the anxiety and depression samples. Second, the DAWBA anxiety band scores did not predict the change in SCARED-CP across treatment. Third, the DAWBA band scores significantly predicted the PARS, a clinician-rated measure of anxiety; however, we did not observe any association between the DAWBA band scores and the change in PARS score across treatment.

Although both the SCARED-CP and MFQ-CP were significantly associated with the DAWBA band scores, the MFQ-CP exhibited a strong association with the DAWBA MDD band score, while the association between the SCARED-CP and the DAWBA anxiety band scores was weak to moderate. This could reflect the high degree of heterogeneity in the anxiety sample, a transdiagnostic grouping of three distinct anxiety disorders, which were not equally represented in our sample. Evaluating anxiety symptoms within specific diagnostic categories likely enables more robust prediction than clustering across disorders [29]. Furthermore, previous studies find that incorporating both self-report and clinician interviews better capture the heterogeneity of internalizing psychopathology and provide more accurate symptom assessment and prediction [30, 31].

As with the SCARED-CP, all DAWBA anxiety band scores significantly predicted the PARS. Relative to other band scores, the relationship between the PARS and the Sep AD band score appeared notably weaker. This is potentially reflective of the low prevalence of Sep AD within our sample. Other studies have also found discrepancies between established self-rated measures and clinician interviews [32, 33]. One study examining the self-reported and clinician-rated versions of the same instrument across novel interventions for depression suggested that each rater contributes distinct and important information for predicting treatment outcomes [31].

In our exploratory analysis, we tested whether the DAWBA band scores, collected at baseline, predicted anxious participants’ response to treatment, as measured by the difference score between participants’ pre- and post-treatment PARS and SCARED-CP. For both measures, none of the three DAWBA band scores (GAD, Sep AD, or Social AD) significantly predicted the change in anxiety across treatment. It is possible that the small sample size in this subset of data constrained our power to detect a predictive effect of the DAWBA band scores on treatment progression. Alternatively, given the DAWBA’s design as a scalable, computer-based diagnostic screening tool administrable by non-expert interviewers, the DAWBA might not possess the granular sensitivity to reliably detect small changes in symptom presentation over relatively short time scales. As established clinical measures collected at baseline are often predictive of symptom progression across treatment when assessed via repeated measurement [17–19], we tested whether PARS and SCARED-CP at baseline predicted the PARS and SCARED-CP difference scores, respectively. As with the DAWBA, neither baseline measurement predicted the change in symptoms across treatment, suggesting overarching sample size constraints and perhaps an inherent complexity in anxiety psychopathology, which may limit accurate prediction over extended timescales.

Although the DAWBA takes advantage of multiple reporters – both parents and children – to make predictions about psychopathology severity via computer algorithms, it remains unclear whether the optimal weighting of parent and child data differs between concurrent prediction of diagnostic risk and prospective prediction of treatment response. Differential weighting of parent and child data may be necessary, especially given the discrepant nature by which parents and children often perceive treatment progression and efficacy [34–36], thereby potentially reducing the DAWBA’s sensitivity to temporal changes in symptomology. Future studies would benefit from exploring the parent- and child-specific DAWBA band scores (rather than the combined, as used in the current study) in conjunction with disorder-specific subscales of established measures to independently predict longitudinal symptom progression across treatment.

To our knowledge, this study is one of the first to examine the DAWBA in relation to established internalizing measures in a sample of treatment-seeking youth both at baseline and across CBT. Importantly, clinicians in the anxiety cohort were masked to the DAWBA when assessing symptom severity. Our results suggest that when collected at baseline, DAWBA band scores are associated with the SCARED-CP, MFQ-CP, and PARS; however, this baseline measurement was not predictive of symptomatology following treatment within the anxiety cohort. Therefore, our findings indicate that DAWBA band scores are relatively predictive of current symptom presentation as per established measures of youth depression and anxiety. However, several limitations are worth noting. First, as our sample comprised only treatment-seeking youth who met diagnostic criteria for a DSM-5 disorder, the associations observed may be reflective of individuals who present with more severe psychopathology and may be less generalizable to subclinical populations. Second, we did not include a clinician-rated measure of depression given sample size constraints of such data across treatment. Third, across all analyses in the anxiety cohort, subjects were aggregated into a transdiagnostic sample spanning three distinct DSM-5 anxiety disorders.

Future studies would benefit from exploring disorder-specific associations between the DAWBA’s three anxiety band scores and subscales of established measures within diagnostically homogenous groups. More precise approaches such as these would allow for more rigorous evaluation of the DAWBA’s anxiety band scores specific to particular symptom clusters. Additionally, assessing the DAWBA’s parent- and child-generated band scores individually in relation to established clinical measures remains important for refining the way in which semi-structured lay interviews differentially weight parents’ and children’s response data to optimize predictions. Finally, future studies should explicitly evaluate the DAWBA band scores with repeated administrations across treatment to more thoroughly assess sensitivity to treatment response.

In conclusion, this is one of the first studies to examine the DAWBA in relation to validated and widely used measures of internalizing psychopathology in a sample of treatment-seeking youth. Principally, our findings suggest that the DAWBA may be an effective tool for screening youth anxiety and depression at relatively transient timescales in relation to established clinical and self-reported measures. However, the DAWBA appeared to be notably limited in predicting anxious participants’ symptom progression across treatment. Despite this, our results suggest some potential for clinical utility in identifying internalizing symptomology among treatment-seeking youth; future studies should further evaluate the DAWBA’s validity and reliability as a scalable tool for mental health screening and assessment.

### Electronic supplementary material

Below is the link to the electronic supplementary material.

Supplementary Material 1

## Data Availability

The data that support the findings of this study will be publicly available, upon acceptance of our manuscript, through NIH-approved repositories.

## Declarations

### Citation recommendation

Amelio & Antonacci et al., (2023)
