# Supplementary material for: Evaluating the development and well-being assessment (DAWBA) in pediatric anxiety and depression
Source: Child Adolesc Psychiatry Ment Health. 2024 Jan 20;18:12. doi: 10.1186/s13034-023-00696-7 (PMC10800067; doi:10.1186/s13034-023-00696-7)
Supplement: Supplementary file 1 — Supplementary Material 1 [file 13034_2023_696_MOESM1_ESM.pdf]

Supplemental Information

Evaluating the Development and Well-Being Assessment (DAWBA) in Pediatric Anxiety and Depression

Paia Amelio[1*], Chase Antonacci[1*], Parmis Khosravi[1], Simone Haller[1], Katharina Kircanski[1], Erin Berman[1], Lisa Cullins[1], Krystal Lewis[1], Mollie Davis[1], Chana Engel[1], Kenneth Towbin[1], Argyris Stringaris[2,3], Daniel S. Pine[1]

[1] Emotion and Development Branch, National Institute of Mental Health, National Institutes of Health, Bethesda, Maryland, USA

[2] Divisions of Psychiatry and Psychology and Language Science, University College London, London, UK

[3] National and Kapodistrian University of Athens, Athens, Greece

[*] Equal contribution

Correspondence to Parmis Khosravi, Emotion and Development Branch, NIMH, 15K North Drive Bethesda, MD 20892, USA. E-mail: parmis.khosravi@nih.gov

Methods

Sample 1 Anxiety Inclusion Criteria

Subjects were included and excluded based on certain diagnostic criteria. Full details on inclusion and exclusion criteria can be found at https://clinicaltrials.gov/study/NCT00018057.

Inclusion criteria

Eligible subjects met the following criteria:

- IQ > 70 (Assessment based on WASI),
- Subjects with anxiety disorder had a current clinically significant or ongoing diagnosis of Social Phobia, Separation Anxiety, Generalized Anxiety Disorder, or panic disorder (based on K-SADS assessment).
- Subjects with mood disorder had a current clinically significant or ongoing diagnosis of depressive symptoms (based on K-SADS assessment).
- Healthy volunteer participants were medication-free and had no current serious medical conditions, based on a review of their medical history.)

Exclusion Criteria

Subjects were excluded based on the following criteria:

- any serious medical condition or condition that interferes with fMRI scanning
- for patients electing medication, any condition that increases the risk of SSRI treatment
- current use of any psychoactive substance
- current suicidal ideation

- current diagnosis of attention deficit hyperactivity disorder (ADHD) of sufficient severity to require pharmacotherapy,
- current diagnosis of Tourette's Disorder, OCD, post-traumatic distress disorder, and conduct disorder.
- past or current history of mania, psychosis, or severe, pervasive developmental disorder
- subjects with recent use of an SSRI, all subjects must have been free of any SSRI-use for at least one month (fluoxetine six months) and must not have been treated with an SSRI for their current depressive episode.

Sample 2 Depressed Inclusion Criteria

Subjects were included and excluded based on certain diagnostic criteria. Full details on inclusion and exclusion criteria can be found at. For full details, please see https://www.clinicaltrials.gov/study/NCT03388606

Inclusion criteria

Eligible subjects met the following criteria:

- IQ > 70
- Youths who meet DSM-5 criteria for Major Depressive Disorder, which included having a current diagnosis of DSM-5 Major Depressive Disorder (within the last six months from assessment), which are:
    - Five or more of the following symptoms have been present during the same 2-week period and represent a change from previous functioning; at least one of the symptoms is either (1) depressed mood or (2) loss of interest or pleasure.

    - Symptoms cause clinically significant distress or impairment in social, occupational/academic, or other important areas of functioning. The episode is not attributable to the physiological effects of a substance or to another medical condition.

Exclusion Criteria

Subjects were excluded based on the following criteria:

- meets criteria for a past or current diagnosis of schizophrenia, schizophreniform disorder, schizoaffective illness, bipolar disorder, Tourette Disorder, more than mild Autism Spectrum Disorder, Anorexia Nervosa or other severe Eating Disorders.
- Major depression or sub-threshold major depressive episode were due to the direct physiological effects of a drug or a general medical or neurological condition by self and parent report.
- Current active suicidal ideation (i.e., presence of intent for engaging in suicidal behaviors)
- any serious medical condition (such as epilepsy or heart disease requiring medication) by self and parent report.
- Past or current diagnosis of a manic or hypomanic episode, major depression), schizophrenia, schizophreniform disorder, schizoaffective illness, Tourette Disorder, or Autism Spectrum Disorder, Anorexia Nervosa or other severe Eating Disorder.

DAWBA Symptom Scales

In the current study, symptom scales for anxiety, depression, internalizing, and externalizing symptoms were generated by collating and summing responses to questions across individual disorders. As the DAWBA adaptively modulates questions presented to participants

based on past responses, symptom scales were generated using only those questions presented to all participants. Symptom scales were then used in analyses assessing the relationship between the DAWBA and established self- , parent- , and clinician-reported measures of symptom severity (SCARED-Child, SCARED-Parent, MFQ-Child, MFQ-Parent, PARS), as symptom scales offer a transdiagnostic and continuous measure of symptom experience captured by the DAWBA. The symptom scales used in all analyses were developed by Jia and colleagues (2020) and included internalizing behaviors (i.e. anxiety and depression) and externalizing behaviors (i.e. Oppositional Defiant Disorder (ODD), Conduct Disorder (CD), Hyperactivity, Inattention, Impulsivity). The anxiety, depression, and internalizing symptom scales were generated from child-reported DAWBA responses, while the externalizing symptom scale was generated from parent-reported responses.

Statistical Analysis

All statistical analyses were performed in R Studio (version 2022.07.1).

1a: SCARED Regression

To assess DAWBA's sensitivity for anxiety diagnosis compared to other clinical assessments, we computed a cross-disorder DAWBA symptom scales for internalizing, externalizing behavior, and anxiety symptomatology (Jia et al., 2020). The DAWBA anxiety symptom scale is an anxiety-specific subset of questions utilized from the internalizing symptom scales. A Pearson product-moment correlation coefficient was performed with DAWBA symptoms scales and SCARED-child total scores and SCARED-parent total scores. In this study, we primarily looked at the baseline measures of the SCARED and ran a logistic regression

analysis and a partial correlation analysis with the DAWBA symptom scales. The analyses included data collected within three months and closest in date to the collection of DAWBA.

1b: MFQ Regression

The same process was used for MFQ as outlined in Statistical Analyses 1a

2a: PARS Regression

The same process was used for PARS as outlined in Statistical Analyses 1a

2b: PARS Across treatment

To assess the DAWBA's ability to predict treatment outcome, we utilized the PARS collected at patients' initial evaluation, pre-exposure treatment, mid-exposure treatment, and post-exposure treatment. We calculated Pearson correlations using these PARS scores across each patient's treatment and both the DAWBA internalizing symptom scale and the anxiety symptom scale. We further evaluated the SCARED-C's and SCARED-P's ability to predict treatment outcome with the PARS using the same Pearson correlations.

STUDY 1: Relationship Between DAWBA & Established Self-Report Instruments

1a: Self-Report Measure: SCARED

The results found that both the DAWBA anxiety symptom scales were strongly associated with the SCARED self-report and parent-report measures (see Table 3). In contrast,

the DAWBA externalizing symptom scale did not predict the self-report and parent-reported SCARED score.

Table 1: DAWBA Symptoms Scale with Self-Report and Clinician-Report

Predictor	*r*	*df*	*t*	Significance
DAWBA Anxiety				
SCARED-C	.80	60	10.34	< .001
SCARED-P	.69	58	7.21	< .001
PARS	.71	61	7.9	< .001
DAWBA Externalizing				
SCARED-C	.11	60	0.84	> .05
SCARED-P	.22	58	1.69	> .05
MFQ-C	.44	198	6.91	< .001
MFQ-P	.48	197	7.74	< .001
PARS	.36	61	2.97	< .005
DAWBA MDD				
MFQ-C	.77	198	6.91	< .001
MFQ-P	.72	197	14.57	< .001

df: degrees of freedom
Significance levels at $\alpha < 0.05$

1b: Self-Report Measure: MFQ

All three DAWBA symptom scales significantly predicted the self- (MFQ-C) and parent-reported MFQ-P (see Table 1).

STUDY 2: Relationship Between DAWBA & Clinician Assessment of Anxiety

2a: Clinician-Report Measure: PARS

The results showed DAWBA anxiety symptom scales strongly predicted the clinician's PARS score (see Table 1).

2b: PARS Across Treatment

The average difference between the PARS composite score from the screening visit to the last treatment visit (CBT 12) was -4.55, SD = 3.75 (Figure 3) was examined. Neither the DAWBA anxiety symptom scale significantly predicted the PARS difference score, DAWBA Anxiety symptom scale and PARS difference Score: β = -0.2, $F(1, 18) = 0.85$, $p > .05$.

The SCARED-C composite score at baseline (n = 17) significantly predicted the PARS difference score. The SCARED-C composite score and PARS difference Score: β = -0.53, $F(1, 15) = 5.75$, $p = .03$.

The SCARED-P composite score at baseline (n = 17) does not significantly predict the PARS difference score. The SCARED-P composite score and PARS difference Score: β = -0.34, $F(1, 15) = 1.92$, $p = .19$.

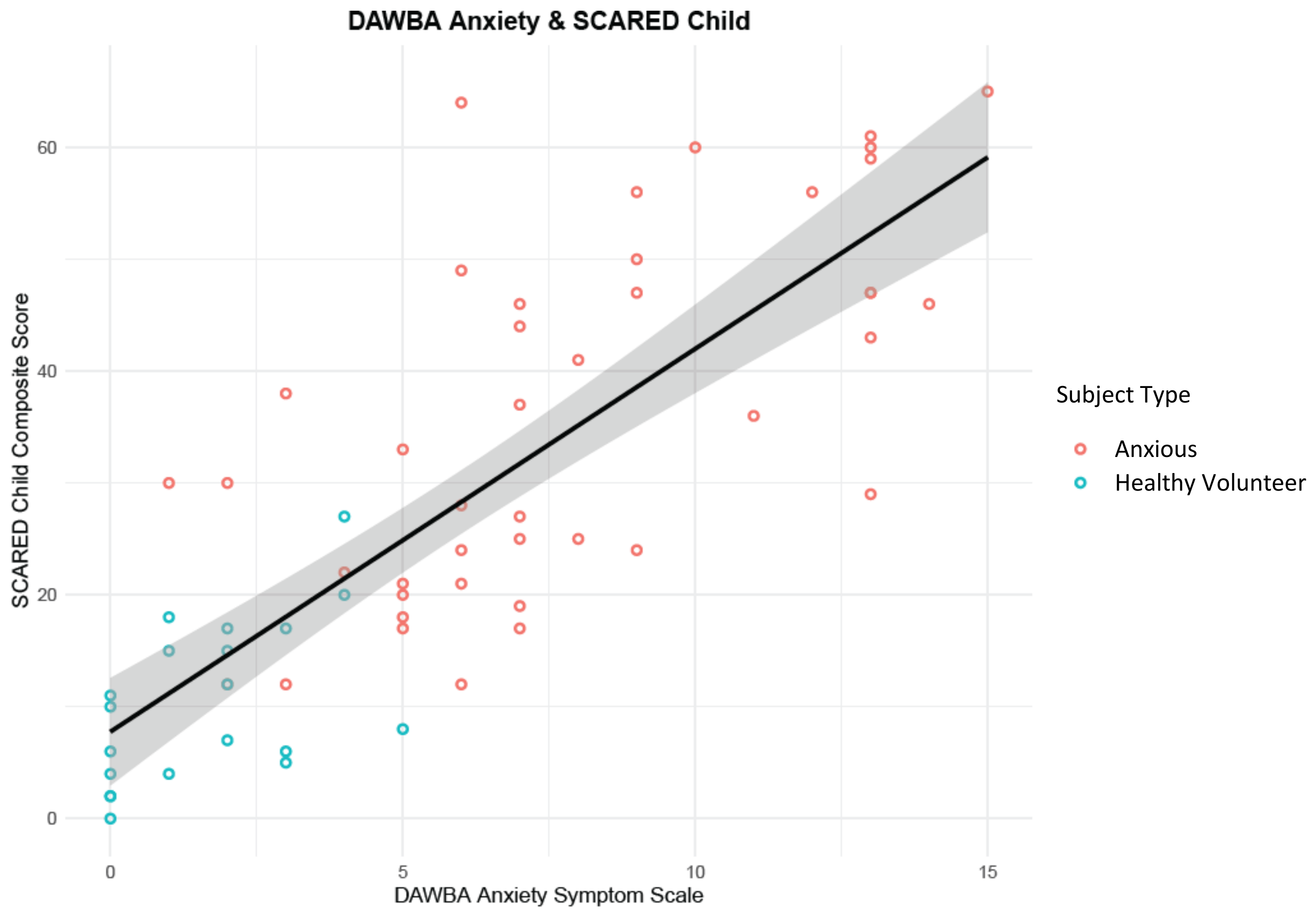

Fig. 1 SCARED-C = self-report Screen for Child Anxiety Related Disorders; SCARED-P = parent-report Screen for Child Anxiety Related Disorders; Anxiety = DAWBA anxiety symptom scale; Internalizing = DAWBA internalizing disorders symptom scale; Externalizing: DAWBA externalizing disorders symptom scale; SCARED-C x Anxiety n = 62; SCARED-C x Internalizing n = 62; SCARED-C x Externalizing n = 62; SCARED-P x Anxiety n = 60; SCARED-P x Internalizing n = 60; SCARED-P x Externalizing n = 60; PARS x Anxiety n = 63; PARS x Internalizing n = 63; PARS x Externalizing n = 63; Blue = healthy volunteers; Red = anxious (GAD, SAD, SoPh)

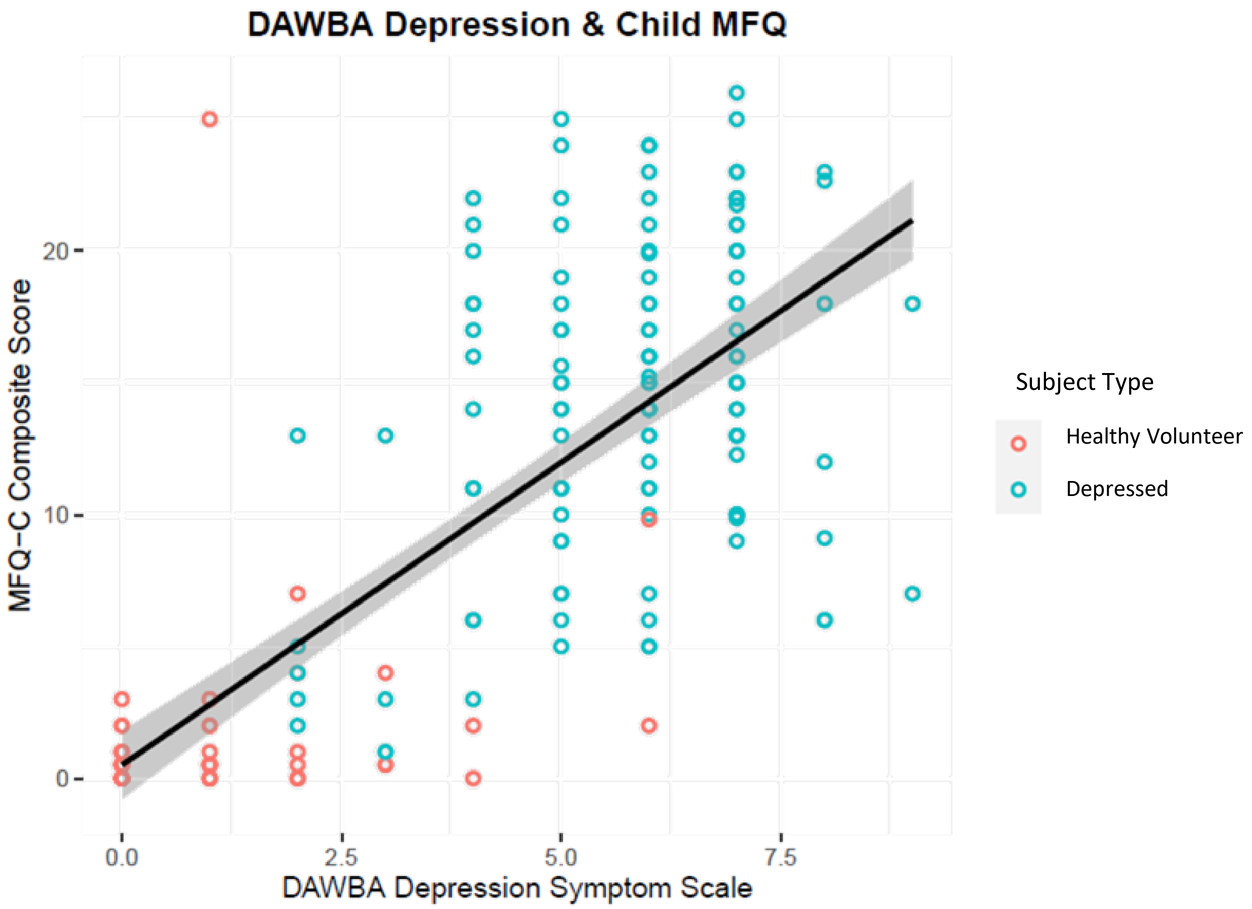

Fig. 2 MFQ-C = self-report Mood and Feelings Questionnaire; MFQ-P = parent-report Mood and Feelings Questionnaire; Depression = DAWBA depression symptom scale; Internalizing = DAWBA internalizing disorders symptom scale; Externalizing: DAWBA externalizing disorders symptom scale. Blue = depressed (MDD); Red = healthy volunteers; MFQ-C x Depression = 200; MFQ-C x Internalizing n = 200; MFQ-C x Externalizing n = 200; MFQ-P x Depression = 199; MFQ-P x Internalizing n = 199; MFQ-P x Externalizing n = 199

Analysis Controlling for Participant Type

An analysis using only data from anxious participants was run to control for participant type. The results showed anxiety and internalizing DAWBA symptom scales positively correlated with a clinician's PARS score, DAWBA anxiety symptom scale: $r = .40$, $t(44) = 2.91$, $p = .006$; DAWBA internalizing symptom scale: $r = .37$, $t(44) = 2.68$, $p = .01$. The DAWBA externalizing symptom scale was weakly and not significantly associated with a clinician's PARS score, $r = .09$, $t(44) = .59$, $p = 0.56$. Further, we found that both the anxiety and internalizing DAWBA symptom scales were positively associated with the SCARED self-report and parent-report measures, DAWBA anxiety symptom scale & SCARED-C: $r = .65$, $t(40) = 5.38$, $p < .001$; DAWBA internalizing symptom scale & SCARED-C: $r = .59$, $t(40) = 4.60$, $p < .001$; DAWBA anxiety symptom scale & SCARED-P: $r = .43$, $t(41) = 3.03$, $p = .004$; DAWBA internalizing symptom scale & SCARED-P: $r = .38$, $t(41) = 2.65$, $p = .01$. In contrast, the DAWBA externalizing symptom scale weakly and insignificantly predicted the self-report SCARED score, DAWBA externalizing symptom scale & SCARED-C: $r = -.22$, $t(40) = -1.41$, $p = .17$, and the parent-report SCARED score, DAWBA externalizing symptom scale & SCARED-P: $r = -.05$, $t(41) = -.33$, $p = .74$.

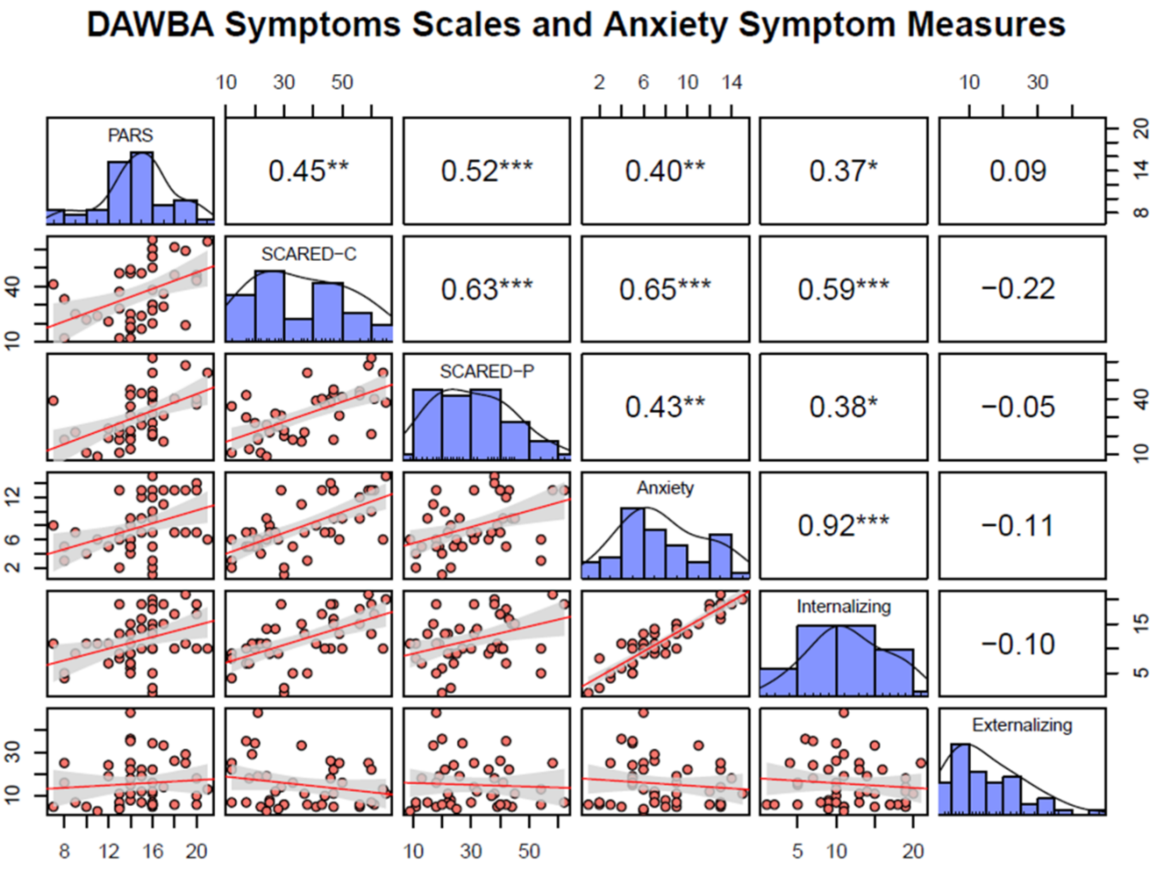

Fig. 3 PARS = Pediatric Anxiety Rating Scale; SCARED-C = self-report Screen for Child Anxiety Related Disorders; Anxiety = DAWBA anxiety symptom scale; Internalizing = DAWBA internalizing disorders symptom scale; Externalizing: DAWBA externalizing disorders symptom scale.
Blue = healthy volunteers; Red = anxious (GAD, SAD, SoPh)

The number of data varied in each correlation. PARS x Anxiety n = 43; PARS x Internalizing n = 43; PARS x Externalizing n = 43
SCARED-C x Anxiety n = 39; SCARED-C x Internalizing n = 39; SCARED-C x Externalizing n = 39
SCARED-P x Anxiety n = 40; SCARED-P x Internalizing n = 40; SCARED-P x Externalizing n = 40
